# Selective electrocatalytic upgrading of nitrogen oxides into hydroxylamine and hydrazine via special routes

**DOI:** 10.1093/nsr/nwaf380

**Published:** 2025-09-10

**Authors:** Shunhan Jia, Xiaofu Sun, Buxing Han

**Affiliations:** Beijing National Laboratory for Molecular Sciences, CAS Laboratory of Colloid and Interface and Thermodynamics, CAS Research/Education Center for Excellence in Molecular Sciences, Center for Carbon Neutral Chemistry, Institute of Chemistry, Chinese Academy of Sciences, China; School of Chemical Sciences, University of Chinese Academy of Sciences, China; Beijing National Laboratory for Molecular Sciences, CAS Laboratory of Colloid and Interface and Thermodynamics, CAS Research/Education Center for Excellence in Molecular Sciences, Center for Carbon Neutral Chemistry, Institute of Chemistry, Chinese Academy of Sciences, China; School of Chemical Sciences, University of Chinese Academy of Sciences, China; Beijing National Laboratory for Molecular Sciences, CAS Laboratory of Colloid and Interface and Thermodynamics, CAS Research/Education Center for Excellence in Molecular Sciences, Center for Carbon Neutral Chemistry, Institute of Chemistry, Chinese Academy of Sciences, China; School of Chemical Sciences, University of Chinese Academy of Sciences, China

## Abstract

This Perspective discusses the design principles of electrocatalytic systems for upgrading nitrogen oxides (NOx) into unconventional products, such as hydroxylamine and hydrazine, while traditional NOx electroreduction produces ammonia or dinitrogen.

Nitrogen oxides (NO_x_), including nitrate (NO_3_^−^), nitrite (NO_2_^−^), nitric oxide (NO), nitrogen dioxide (NO_2_) and nitrous oxide (N_2_O), are major pollutants in wastewater and gases, posing serious environmental and health hazards [1]. However, these species are also nitrogen sources with significant potential for sustainable chemical manufacturing. As illustrated in Fig. 1a, electrochemical NO_x_ reduction uses green electricity as the electron source and water (H_2_O) as the proton donor, enabling ambient conversion of NO_x_ into nitrogenous products such as ammonia (NH_3_) and nitrogen (N_2_) [2]. Both gaseous and aqueous NO_x_ can serve as renewable feedstocks with distinct application domains. Gas-phase NO_x_ is better suited for point-source abatement, while liquid-phase NO_x_ enables wastewater valorization. In addition, the N≡N triple bond in molecular N_2_ is exceptionally stable, making its electrochemical activation highly energy-demanding and kinetically unfavorable. In contrast, the N–O bonds in NO_x_ species possess significantly lower bond energies and can be more readily cleaved to generate reactive intermediates such as *NO and *NH_2_. Consequently, NO_x_ offers a more promising and energetically favorable nitrogen source for electrosynthesis. Nevertheless, unconventional products like hydroxylamine (NH_2_OH) and hydrazine (N_2_H_4_), which hold significant industrial importance and economic worth, have garnered relatively limited attention [3]. NH_2_OH is widely used in agrochemicals, pharmaceuticals and fine chemicals, while N_2_H_4_ serves as an essential propellant, fuel and synthetic intermediate. NH_2_OH and N_2_H_4_ are traditionally synthesized via multi-step thermochemical routes that are energy-intensive and generate secondary wastes. Electrocatalytic upgrading of NO_x_ to NH_2_OH and N_2_H_4_ thus provides not only an opportunity for pollutant remediation under mild and sustainable conditions, but also a pathway to replace conventional processes with greener, electricity-driven alternatives.

**Figure 1.**
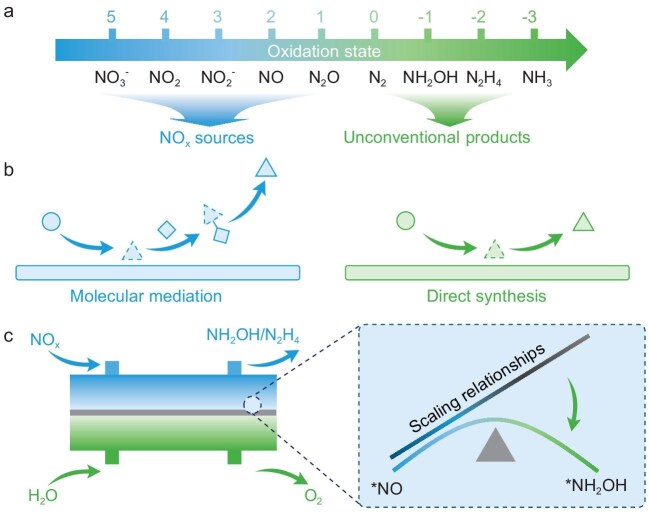
Conceptual framework for electrocatalytic NO_x_ upgrading to unconventional products. (a) Oxidation state diagram of nitrogen species in NO_x_ electroreduction. (b) Molecular mediation (left) and direct synthesis (right) strategies for NO_x_ conversion to unconventional products. (c) Schematic illustration of an electrochemical system for converting NO_x_ into NH_2_OH/N_2_H_4_ in the future.

Selective synthesis of NH_2_OH and N_2_H_4_ through NO_x_ electroreduction remains highly challenging due to the complex and unstable nature of nitrogenous intermediates, which are prone to over-reduction or undesired side reactions. For example, NH_2_OH is often further reduced to NH_3_ due to its strong spontaneous adsorption on catalyst surfaces and slow desorption into the electrolyte [4]. Likewise, the pathways for generating N_2_H_4_ are commonly outcompeted by N−N coupling routes, and they are thermodynamically more favorable for the formation of N_2_. This is due to the easy coupling of intermediates that come from *NO and the reduction of multi-nitrogen species such as *NONH_2_, *N_2_O or *H_2_N_2_O_2_. These multi-nitrogen species quickly break down into N_2_ [5].

To overcome these limitations, two main strategies have been developed, as shown in Fig. 1b. One approach involves using molecular mediation (Fig. 1b, left). In this case, mediator molecules create reversible interactions with nitrogenous intermediates to control their reactivity. Organic mediators like ketones have been effectively employed to capture *NH_2_OH and convert it into stable oximes, preventing its over-reduction [4]. Recent work has shown that ketone-mediated pathways facilitate selective NH_2_OH formation from nitrate by stabilizing *NH_2_OH intermediates through spontaneous condensation into oxime species, which can then be hydrolyzed under mild conditions to regenerate NH_2_OH. Beyond molecular mediation, integrating electrocatalytic NH_2_OH production with downstream reactions using NH_2_OH as an essential intermediate to synthesize other chemicals could enhance both practicality and economic viability by reducing the need for isolation and enabling direct utilization of the NH_2_OH intermediate. Similarly, ketone-mediated synthesis of N_2_H_4_ offers an avenue to circumvent the thermodynamic and kinetic obstacles associated with the direct NH_3_ oxidation [5,6]. In this route, ketones react with NH_3_ or its surrogates to form imine intermediates, enabling controlled N–N bond formation. Notably, benzophenone imine has been identified as an effective surrogate for facilitating oxidative coupling and selective hydrazine generation. The hydrolysis of the formed azine leads to the regeneration of the mediator and the liberation of N_2_H_4_, thus completing a redox catalytic cycle. This strategy minimizes side reactions and decouples electron transfer from the formation of labile N–N bonds. Consequently, it significantly enhances the selectivity and control in the reaction process.

The second strategy focuses on direct electrochemical routes (Fig. 1b, right). Many catalytic systems have enabled the selective reduction of NO_x_ to NH_2_OH and N_2_H_4_. Single-atom catalysts (SACs) based on transition metals such as Zn, Fe and Co can fine-tune the adsorption configurations of intermediates like *NO and *NH_2_OH, promoting NH_2_OH formation while suppressing over-reduction to NH_3_ [7,8]. In particular, SACs have been demonstrated to stabilize the N–O bond via linear adsorption of *NO, achieving high NH_2_OH selectivity. In contrast, bridge adsorption generally leads to bond cleavage and the subsequent formation of NH_3_ [9]. Theoretical studies reveal that the interaction between Fe 3d orbitals and selectivity-determining intermediates (SDIs), such as *NO and *NH_2_OH, plays a critical role in guiding product outcomes. In addition, Ru-based molecular catalysts have demonstrated excellent performance in the selective electrooxidation of NH_3_ to N_2_H_4_, avoiding overoxidation to N_2_ [10]. Complexes such as CSU-1, CSU-2 and CSU-3 exhibit tailored coordination environments and redox properties that promote bimolecular N–N coupling through Ru(II)-aminyl and Ru(III)-iminyl radicals. For instance, CSU-2 achieves a turnover number (TON) of 5735 for N_2_H_4_ over 24 h under mild conditions, significantly outperforming previous molecular systems. To clarify the distinct features of these two electrocatalytic strategies, we provide a comparative summary in Table 1.

**Table 1.** Comparison of molecular mediation and direct synthesis strategies for NO_x_ upgrading.

Strategy	Advantages	Disadvantages
Molecular mediation	1. Enhances selectivity via intermediate stabilization 2. Bypasses kinetic and thermodynamic bottlenecks 3. Enables redox cycling of mediators	1. Requires additional mediator synthesis and separation 2. Limited mediator–catalyst compatibility
Direct synthesis	1. Avoids external additives 2. More streamlined and scalable process 3. Well-defined active sites	1. Suffers from scaling relations between intermediates 2. Prone to over-reduction or undesired pathways

Despite these advances, further progress in catalyst innovation, electrolyte design, reactor engineering and process integration is essential to achieve practical NO_x_-to-NH_2_OH/N_2_H_4_ conversion (Fig. 1c). Although existing catalysts succeed in stabilizing intermediates and offer well-defined adsorption environments, their catalytic activity and selectivity are fundamentally hindered by scaling relationships among adsorbed species. These relationships typically arise due to the uniform electronic structures at catalytic sites. It leads to a trade-off situation, in which improving the binding of one intermediate usually has an unfavorable impact on the binding of the others. Circumventing these scaling relationships will require the design of catalysts with asymmetric geometries, tunable electronic environments or cooperative multi-site architectures. In addition to activity and selectivity, the long-term stability of catalytic systems remains a critical challenge for practical NO_x_ upgrading. Strategies such as stabilizing single-atom sites, designing robust molecular coordination environments and optimizing electrolytes will be essential to ensure durability under continuous electrolysis. Electrolyte composition strongly influences reaction selectivity by governing proton availability, interfacial electric fields, and intermediate stabilization; thus, tailoring electrolyte species and additives represents an important strategy for optimizing NO_x_ upgrading pathways. Beyond catalysts and electrolytes, the rational design of electrochemical devices is crucial. For gaseous NO_x_ species like NO and N_2_O, their low solubility in aqueous media necessitates the creation of three-phase interfaces for efficient mass and charge transfer. Gas-diffusion electrodes (GDEs) provide such environments, facilitating high current density operation, reducing diffusion barriers and suppressing hydrogen evolution. At the system level, utilizing membrane electrode assemblies (MEAs) in electrochemical stacks offers a scalable pathway for industrial deployment. Integration with upstream NO_x_ generation/capture technologies, renewable energy sources and downstream product separation will be important for the development of a sustainable and economically viable process. Moreover, because NH_2_OH and N_2_H_4_ are energetic compounds, process safety is a critical factor for practical application. Optimized reaction conditions, selective catalysts and flow-based reactor designs can minimize product accumulation, suppress side reactions, and thereby mitigate risks of decomposition in large-scale synthesis.

In conclusion, electrocatalytic upgrading of NO_x_ into unconventional products such as NH_2_OH and N_2_H_4_ represents a promising avenue for transforming environmental pollutants into valuable chemicals under mild, sustainable conditions. This approach can not only utilize green electricity and H_2_O but also expands the chemical product space beyond conventional outputs like NH_3_ and N_2_. Recent advancements in both mediator-assisted and direct electrocatalytic pathways have significantly enhanced reaction selectivity by stabilizing reaction intermediates. Concurrent progress in catalyst design and electrochemical device development is laying the groundwork for efficient, scalable and integrated NO_x_ upgrading systems. Beyond NH_2_OH and N_2_H_4_, NO_x_ electroreduction also offers opportunities for generating other unconventional products, which may open new avenues for sustainable nitrogen chemistry. These insights shed light on strategies for converting unstable intermediates into desired products and may also inspire the sustainable synthesis of other value-added chemicals with distinctive chemical reactivity.
